# Dynamics between insight and medication adherence in first-episode psychosis: Study of 3-year trajectories

**DOI:** 10.1192/j.eurpsy.2022.2305

**Published:** 2022-08-15

**Authors:** Julien Elowe, Julie Ramain, Alessandra Solida, Philippe Conus, Philippe Golay

**Affiliations:** 1 Service of Adult Psychiatry North-West, Department of Psychiatry, Lausanne University Hospital (CHUV), Chemin des Chaux, 1196 Prangins, Switzerland; 2 Service of General Psychiatry, Treatment and Early Intervention in Psychosis Program (TIPP-Lausanne), Lausanne University Hospital (CHUV) and University of Lausanne (UNIL), Lausanne, Switzerland; 3 Department of Adult Psychiatry II, Centre Neuchâtelois de Psychiatrie, Site de Préfargier, Marin-Epagnier, Switzerland; 4 Community Psychiatry Service, Department of Psychiatry, Lausanne University Hospital (CHUV), Lausanne, Switzerland; 5 Institute of Psychology, Faculty of Social and Political Science, University of Lausanne, Lausanne, Switzerland

**Keywords:** First-episode psychosis, insight, medication adherence, specialized early psychosis program

## Abstract

**Background:**

While specialized early intervention programs represent the gold standard in terms of optimal management of first-episode psychosis (FEP), poor medication adherence remains a predominant unmet need in the treatment of psychosis. In this regard, an interaction between insight and adherence in FEP patients has been hypothesized but has been challenged by multiple pitfalls.

**Methods:**

Latent profile analysis and trajectory modeling techniques were used to evaluate insight and adherence of 331 FEP patients engaged at the beginning, middle, and end of a 3-year specialized early psychosis program. A Bayesian model comparison approach was used to compare scores of clinical, functional, and socioeconomic outcomes at the end point of the study.

**Results:**

Nearly one-third of the patients maintain a high level of insight and adherence during the entire program. At the end of the 3-year follow-up, more than three-quarters of patients are considered adherent to their medication. Patients with low levels of insight and adherence at the beginning of the program improve first in terms of adherence and then of insight. Furthermore, patients with high levels of insight and adherence are most likely to reach functional recovery and to experience an increase in environmental quality of life.

**Conclusions:**

Latent FEP subpopulations can be identified based on insight and adherence. Medication adherence was the first variable to improve, but a gain in insight possibly plays a role in the reinforcement of adherence.

## Introduction

It is widely accepted that the onset of a first-episode psychosis (FEP) requires rapid identification of symptoms and early management consisting of appropriate antipsychotic treatment and personalized psychosocial and psychoeducational interventions [1]. Such specialized interventions intend to maximize clinical and functional prognostic outcomes of patients during the critical period, that is, the first 2–5 years after the onset of psychosis [2]. If reducing the duration of untreated psychosis is a priority goal, medication adherence also plays a major role and remains one of the most important factors associated with symptomatic remission [3]. Indeed, nonadherence to medication in the treatment of schizophrenia can have a negative impact at the clinical level, with more frequent relapses, later remissions, the development of symptoms more resistant to treatment, a lower level of functioning, a lower quality of life and higher suicide rates, as well as at the socioeconomic level, with higher costs to the healthcare system [4–9]. Similar findings are seen in FEP studies [10–16]. Many factors implicated in poor medication adherence in FEP have been described elsewhere, including patient-related, environment-related, medication-related, and illness-related factors [17].

While the concept of *compliance to treatment* has gradually been replaced, implying the patient’s passive attitude toward his treatment, *adherence to treatment* refers to a more active implication of the patient [18]. In this respect, nonadherence is considered when only some or none of the prescribed antipsychotic medication is taken. In patients with schizophrenia, the mean rate of medication nonadherence approximates 50% [7] and tends to increase after hospital discharge: nearly 25% within 7–10 days, 50% 1 year later, and up to 75% within 2 years [19, 20]. In FEP patients, rates of medication nonadherence can also reach high levels, that is, 30–40% 6 months after the onset of the episode and 50% at 1-year follow-up [21, 22]. In this respect, the implementation of specialized early intervention programs has a direct positive effect on the rate of nonadherence, usually between 15 and 20% in the first 2 years of follow-up [23, 24].

Some authors have suggested a possible interaction between insight and treatment adherence in FEP patients [25, 26]. Clinical insight usually comprises three dimensions: awareness of the illness and symptoms, the need for treatment, and understanding the psychosocial difficulties attributed to the illness [27]. Poor clinical insight into psychosis has been repeatedly identified as a major factor associated with treatment nonadherence [7, 28–31], both at the onset of the psychotic episode and after 1 year of follow-up [21, 32]. The level of insight thus seems to be a good predictor of antipsychotic medication adherence, level of functioning, and global clinical outcome at 1-year follow-up [25]. However, not all studies reach this conclusion [33]. These contradictory results may be partly explained by the cross-sectional or longitudinal nature of the study design. While cross-sectional studies generally find a positive correlation between good insight and better medication adherence [34, 35], longitudinal studies reach contrasting conclusions: no association between the two [36, 37] or a correlation only in the need for treatment [38].

One possible explanation of this discrepancy could be the complexity of conceptualizing insight given its dynamic features [26]. Moreover, the variability of the assessment’s methods of both insight and medication adherence does not allow a standardization of the results [19]. Finally, based on the existing literature, psychiatrists are faced with *the chicken or the egg* causality dilemma: Are adherent patients more likely to increase their insight during follow-up or does a high level of insight at baseline allow for good adherence? This issue remains largely unanswered.

In this study, we comprehensively and prospectively explored the association between dynamic change in insight and change in medication adherence in FEP patients engaged in a 3-year specialized early psychosis program in order to fathom the potential interlacing between these two constructs in time. We addressed the following questions: (a) Is it possible to identify subpopulations within a population of FEP patients based on the two variables of insight and medication adherence? (b) If so, what trajectories do these subpopulations follow over the 3 years of follow-up? (c) How do these different subpopulations differ regarding certain clinical, functional, and socioeconomic outcomes at the end of the 3-year program?

## Methods

### Study design

A specialized outpatient early psychosis program (Treatment and early Intervention in Psychosis Program or TIPP) has been designed and implemented at the Department of Psychiatry in Lausanne University Hospital, Switzerland in 2004 [39], consisting of a 3-year individual treatment by a psychiatrist and a case manager. The TIPP program favors a bio-psycho-social perspective, and as such provides treatment that includes psychotherapy, psychoeducation, family support and therapy, cognitive assessment and remediation, social support, supported employment, psychological interventions for cannabis use, and pharmacological treatment. In line with international guidelines, atypical antipsychotics are first-line pharmacological treatment with a prospective monitoring of any side effects [39]. Case managers meet patients frequently over the treatment period, which provides the framework to establish a trusting relationship, where extensive knowledge of patients’ history can be gathered. At baseline, a specially designed questionnaire [40] is completed by the case managers, and additional relevant information is eventually during follow-up. The main areas assessed are sociodemographic elements, past medical and psychiatric history, insight into the illness, substance use, adherence to medication, exposure to life events, and global functioning. Throughout the program, follow-up assessments are made at 2, 6, 12, 18, 24, 30, and 36 months, respectively. Symptomatology is assessed by trained psychologists and psychiatrists within the same deadlines.

### Participants

Patients are referred to the program by the hospital, general practitioners, social professional networks, or families. Therefore, the study sample is representative of the entire population of patients with FEP who need specialized psychiatric treatment. Inclusion criteria to the program are age between 18 and 35, residence in the catchment area, and meeting criteria for psychosis according to the “Psychosis threshold” subscale of the Comprehensive Assessment of At Risk Mental State scale [41]. Participants who received antipsychotic medication for a total duration of more than 6 months, or those with mental retardation (IQ < 70), or displaying psychosis secondary to substance use or organic brain disorders are referred to other treatment programs.

This study was approved by the Human Research Ethics Committee of the Canton de Vaud (protocol #2020–00272) and complied with the Declaration of Helsinki on medical protocol. All parents were informed on study details and provided consent prior to the inclusion. The data generated by the follow-up of all patients were used in the study if they did not explicitly object to the use of their data for research purposes. Only four patients refused their clinical data to be used for research. Patients included in this study started the program between January 2004 and March 2019.

### Measurements

For each participant, insight was assessed at baseline and follow-up by the same case manager using a Likert type scale [42]. Insight was considered either absent (0), partial (1), or totally present (2). This rating was substantially negatively correlated (ρ ranging between −0.547 and −0.414) with the G12 PANSS item (lack of judgment and insight) at all follow-up assessments, suggesting good convergent validity [43, 44].

Information about medication adherence was collected from the participants and their family. Patients taking their medication between 75 and 100%, between 25 and 75%, and less than 25% of the time are considered totally adherent (score 2), partially adherent (score 1), and nonadherent to medication (score 0), respectively.

### Outcomes

For the outcome measures, we considered scores at discharge, that is, at 36 months follow-up, or 30 if not available. We used Andreasen’s criteria (score ≤ 3) to determine symptomatic recovery which is based on eight items of the PANSS (delusion, unusual thought content, hallucinatory behavior, conceptual disorganization, mannerisms, blunted affect, social withdrawal, lack of spontaneity) [45]. The general functional level was assessed with the Global Assessment of Functioning (GAF) [46] that considers social and occupational levels, and the impact of symptomatology. A GAF score above 60 defined general functional recovery. The premorbid functional level was assessed with the Premorbid Adjustment Scale (PAS) [47]. A PAS score at or below the premorbid rating on four of the five items of the general PAS defined functional recovery [48]. Quality of life at discharge was assessed with the World Health Organization Quality of Life scale [49]. This 5-point likert scale measures satisfaction with life and self-esteem through 26 self-rated items ranging from 1 (low satisfaction) to 5 (high satisfaction). The assessment of independent living recovery (head of household/living alone, with partner, or with peers/living with family with minimal supervision) was carried out using the Modified Vocational Status Index. The assessment of working recovery (paid or unpaid full- or part-time employment/being an active student in school or university/head of household with employed partner (homemaker)/full or part-time volunteer) was carried out using the Modified Location Code Index Independent living [50]. Insight recovery was defined as full insight at discharge.

### Statistical analysis

We first performed a latent profile analysis (LPA) for the beginning, middle, and end of the program. We used insight and adherence scores as indicators. To guarantee model statistical identification and cope with data that could be missing from one assessment, each LPA was estimated using adherence and insight scores of two neighboring assessment (2 and 6 months, 18 and 24 months, and 30 and 36 months) with measurement invariance imposed across the two insights, respectively, the two adherence indicators. The best solution was determined using the Bayesian information criterion (BIC) coefficient, which balances model fit and model complexity [51].

In a second step, we performed a latent transition analysis (LTA). Given the meaning of the classes was very close between each three LPAs, we also imposed longitudinal measurement invariance in the LTA model. While reducing the total number of model parameters, it also ensured the meaning of the classes would stay the same between the beginning, middle, and the end of the program, thus facilitating interpretation.

Finally, in order to compare scores from the different classes at the end point of the study, we used a Bayesian model comparison approach. This represents an alternative to the problem of multiple comparisons and allows evaluating the support for the null hypothesis [52–54]. The first model was the homogeneous model (1, 2, 3, 4), stating that the four groups did not differ and were issued from the same distribution. It corresponds to the null hypothesis in the classical statistical framework. Another model was the heterogeneous model: (1) (2) (3) (4) (i.e., all the groups were different from each other and were issued from four different distributions). All other possible combinations, which adds up to 15—for instance (1, 2, 3), (4) or (1, 3), (2, 4)—were estimated. For continuous variables, the best possible Gaussian model (μ, σ^2^) was determined by using the BIC [51]. For nominal variables, the best multinomial model was determined using the exact likelihood with a uniform prior on all parameters [54]. An equal prior probability of 1/15 was assumed for all models so that no model was favored. The Bayes factor was also computed [55] and provided a comparison between the best model and the homogenous model. A Bayes factor of 4 indicates that the best model was 4 times more likely to be true than the homogenous model.

All statistical analyses were performed using the Mplus statistical package, version 8.3, IBM SPSS, version 25, and the Bayes R2STATS group models calculator [56].

## Results

Eighty-eight patients for whom there was no indication for medication at some point during follow-up were excluded. The final sample consisted of 331 patients (mean age = 24.5; SD = 4.53) and included mainly males (67.4%). Among these, 62.5% met diagnostic criteria for schizophrenia, 10.3% for schizoaffective disorder, 9.4% for schizophreniform or brief psychotic disorder, 6.6% for bipolar disorder, 2.1% for depression with psychotic features, and 4.2% for other psychotic disorders. The mean duration of illness was 1.04 years (SD = 2.40). The different LPA solutions are presented in Table 1. 22.0% of data was missing. The covariance coverage ranged from 88.8 and 59.5% for any pair of variables, which we deemed adequate. A three-class solution was chosen for the first LPA (beginning of the program) based on its lowest BIC and clinical interpretability. Adding an extra-class yielded a higher BIC coefficient and resulted in an empty class. The first class consisted of patients with high insight and high adherence, labeled *high* in the tables. The second class consisted of patients with low insight and low adherence, labeled *low* in the tables. The third class consisted of patients with low insight and high adherence.

**Table 1.** Characteristics of the three class latent class analysis solutions.

	Number of classes	Entropy	BIC
Beginning (2–6 months)	1	—	2,536.860
	2	0.897	2,176.097
	**3**	**0.877**	**2,108.989**
	4^a^	0.813	2,126.199
Middle (18–24 months)	1	—	2,215.958
	2	0.931	1,868.428
	3	0.813	1,813.694
	**4**	**0.920**	**1,778.280**
	5^a^	0.931	1,795.227
End (30–36 months)	1	—	1,996.930
	2	0.964	1,584.545
	3	0.900	1,511.827
	**4**	**0.955**	**1,474.629**
	5	0.954	1,489.453

*Note:* The best class was determined on the basis of the lowest BIC coefficient and is indicated in bold.

Abbreviation: BIC, Bayesian information criterion.

a one class is empty.

For the LPA performed at the middle of the program, BIC indicated a four-class solution. It consisted of three classes similar to those observed in the first LPA with the addition of a fourth class with high insight and low adherence.

The LPA performed at the end of the program also suggested a four-class solution with a very similar interpretation.

**Figure 1.**
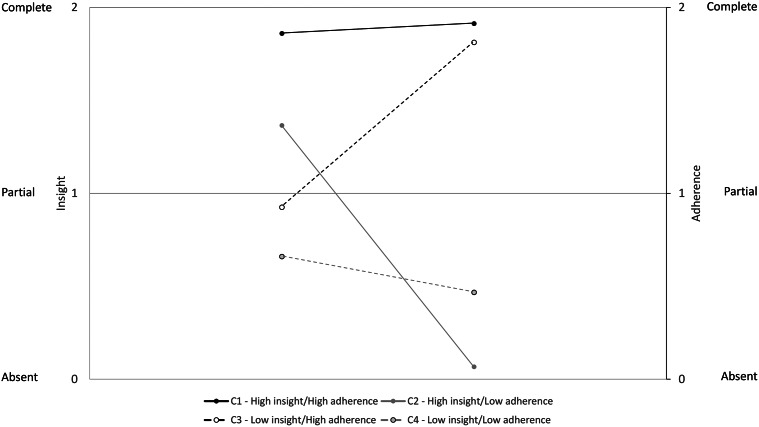
Characteristics of the four longitudinally invariant classes.

Finally, the LTA was estimated. The characteristics of the four longitudinally invariant classes are represented in Figure 1.

Insight and adherence trajectories over the 3-year program are presented in Table 2. Results suggest that about 31% of patients display full insight and adherence throughout the program. At the end, 57.4% of patients have high insight and high adherence. Nevertheless, insight and adherence will remain low during the entire 3-year follow-up in 10.6% of patients and 19.3% of the total sample will end the program with low insight and low adherence. Results indicate that high adherence with low insight is more frequent than good insight with low adherence.

**Table 2.** Insight and adherence trajectories over 3 years of treatment (*N* = 331).

Beginning	Middle	End	*N*	%
High	High	High	102	0.30816
Low insight/High adherence	High	High	42	0.12689
Low	Low	Low	35	0.10574
Low insight/High adherence	Low insight/High adherence	Low insight/High adherence	33	0.09970
Low	High	High	17	0.05136
Low insight/High adherence	Low insight/High adherence	High	16	0.04834
Low	Low insight/High adherence	Low insight/High adherence	14	0.04230
Low insight/High adherence	Low	Low	11	0.03323
Low	High insight/Low adherence	High insight/Low adherence	8	0.02417
Low	Low insight/High adherence	Low	7	0.02115
Low	Low	Low insight/High adherence	7	0.02115
Low insight/High adherence	Low insight/High adherence	Low	6	0.01813
Low	Low insight/High adherence	High	5	0.01511
Low	Low	High	4	0.01208
High	High	Low insight/High adherence	3	0.00906
High	High insight/Low adherence	High insight/Low adherence	3	0.00906
High	High insight/Low adherence	Low	3	0.00906
Low insight/High adherence	High	Low insight/High adherence	3	0.00906
Low insight/High adherence	Low	High	3	0.00906
Low	High	Low insight/High adherence	2	0.00604
Low	High insight/Low adherence	Low	2	0.00604
High	Low insight/High adherence	High	1	0.00302
High	Low insight/High adherence	Low insight/High adherence	1	0.00302
Low	High	High insight/Low adherence	1	0.00302
Low insight/High adherence	High	High insight/Low adherence	1	0.00302
Low insight/High adherence	Low	Low insight/High adherence	1	0.00302

*Note:* High, High insight/High adherence; Low, Low insight/Low adherence. The trajectories are presented according to their frequency of occurrence in the cohort.

Transitions between the beginning, the middle, and the end of the program are presented in Table 3.

**Table 3.** Transition matrices between the beginning, the middle, and the end of the program.

				Middle of the program			
				High	High insight/Low adherence	Low insight/High adherence	Low
Beginning of the program		High	Count	**105**	6	2	0
			% within beginning	**92.9%**	5.3%	1.8%	0.0%
		Low	Count	20	10	26	**46**
			% within beginning	19.6%	9.8%	25.5%	**45.1%**
		Low insight/High adherence	Count	46	0	**55**	15
			% within beginning	39.7%	0.0%	**47.4%**	12.9%

			End of the program			
			High	High insight/Low adherence	Low insight/High adherence	Low
Middle of the program	High	Count	**161**	2	8	0
		% within middle	**94.2%**	1.2%	4.7%	0.0%
	High insight/Low adherence	Count	0	**11**	0	5
		% within middle	0.0%	**68.8%**	0.0%	31.3%
	Low insight/High adherence	Count	22	0	**48**	13
		% within middle	26.5%	0.0%	**57.8%**	15.7%
	Low	Count	7	0	8	**46**
		% within middle	11.5%	0.0%	13.1%	**75.4%**

			End of the program			
			High	High insight/Low adherence	Low insight/High adherence	Low
Beginning of the program	High	Count	**103**	3	4	3
		% within beginning	**91.2%**	2.7%	3.5%	2.7%
	Low	Count	26	9	23	**44**
		% within beginning	25.5%	8.8%	22.5%	**43.1%**
	Low insight/High adherence	Count	61	1	**37**	17
		% within beginning	52.6%	0.9%	**31.9%**	14.7%

*Note:* Cells in bold indicates stability (patients who stayed in the same class between the two assessments).

Results of classes transitions between the beginning and the middle of the program suggest that patients who start with high insight and adherence tend to keep their insight and adherence high. Patients with both poor insight and adherence tend to improve first with adherence and only later regarding insight. Patients who start with low insight and high adherence are likely to gain insight or remain stable but keep a high level of adherence.

Transitions between the middle and the end of the program suggest more stability between identical classes at different time points. Patients starting in the high insight and adherence class and worsening are more likely to lose complete adherence than insight. Again, patients with low adherence and insight at the beginning of treatment were likely to improve first regarding adherence and then regarding insight.

Finally, transition between the beginning and the end of the program confirms the stability of patients with high insight and adherence throughout the 3 years (91.2%, *n* = 103). Interestingly, 56.8% of patients who start with low insight and adherence are likely to improve, although a gain in adherence is more likely than a gain in insight.

Comparison of outcomes between groups with varying levels of insight and adherence at the end of the program is presented in Table 4. Patients with high insight and adherence at the end of the treatment are more likely to achieve functional recovery and better environmental quality of life. Other dimensions of quality of life were unaffected by insight and adherence. Patients with good insight but poor adherence seemed to be most at risk in not recovering symptomatically. Return to employment was particularly challenging for the group with both poor insight and adherence. Independent living was highest for patients with high insight regardless of adherence and was worst for patients with low insight and high adherence. Premorbid adjustment recovery was more likely for patients with high adherence and not related to insight, although this model was only marginally more likely than the null hypothesis.

**Table 4.** Comparison of outcomes between groups of insight/adherence levels at the end of the program.

	(1) High insight/High adherence (*n* = 190)	(2) High insight/Low adherence (*n* = 13)	(3) Low insight/High adherence (*n* = 64)	(4) Low insight/Low adherence (*n* = 64)	Best model^a^	Bayes factor against null hypothesis^b^	Probability of the model to be true^c^
Symptomatic recovery, % (*n*)	50.0 (48)	0.0 (0)	37.5 (9)	27.3 (3)	(1, 3, 4), (2)	3.3,701	0.1,440
General functional recovery, % (*n*)	49.0 (74)	25.0 (3)	25.9 (15)	20.5 (9)	(1), (2, 3, 4)	1,188.8102	0.4,493
Premorbid adjustment recovery, % (*n*)	49.0 (50)	33.3 (3)	39.1 (18)	27.6 (8)	(1, 3), (2, 4)	1.2,976	0.1,386
Independent living recovery, % (*n*)	62.0 (88)	90.9 (10)	37.5 (21)	51.2 (21)	(1, 4), (2), (3)	80.2,743	0.3,009
Working recovery, % (*n*)	31.7 (45)	36.4 (4)	23.2 (13)	12.2 (5)	(1, 2, 3), (4)	2.9,981	0.2,220
Quality of life, M (SD)							
*Quality of physical health*	24.87 (4.32)	24.33 (2.89)	26.46 (5.32)	25.25 (5.38)	(1, 2, 3, 4)	1.0000	0.4,278
* Quality of psychological aspects*	21.17 (3.56)	20.33 (5.03)	22.38 (4.06)	21.00 (4.40)	(1, 2, 3, 4)	1.0000	0.4,400
* Quality of social relationships*	10.69 (2.02)	9.33 (1.53)	10.35 (2.90)	10.13 (2.25)	(1, 2, 3, 4)	1.0000	0.4,363
* Quality of environment*	30.50 (5.24)	24.67 (2.89)	25.95 (7.04)	24.75 (9.74)	(1), (2, 3, 4)	22.3,733	0.4,947

a Based on BIC coefficient;

b Bayes factor comparing the best model to the homogeneous model (1, 2, 3, 4);

c Compared to all possible models.

## Discussion

In this study, our goal was (a) to identify subpopulations within a population of FEP patients based on the two variables of insight and medication adherence, (b) to describe what trajectories do these subpopulations follow over the 3 years of follow-up, and (c) to verify how do these different subpopulations differ regarding clinical, functional, and socioeconomic outcomes at the end of the 3-year program. We made the following observations: First, patients with low levels of insight and medication adherence at the beginning of the program tend to improve during follow-up, first in terms of adherence and then of insight. Second, nearly one-third of the total sample of patients (31%) maintain a high level of insight and adherence during the entire program and more than half of them (57.4%) end the program following the same pattern. Furthermore, over 76% of patients are considered adherent to their medication at the end of the program. Third, more than 90% of patients who display high levels of insight and adherence when entering the program remain so over the 3-year follow-up. Fourth, at the end of the follow-up, one patient out of five has a low level of insight and adherence. Finally, patients with high levels of insight and adherence are most likely to reach functional recovery and to experience an increase in environmental quality of life.

Most cross-sectional studies conclude that a high level of insight is correlated with adequate medication adherence [21, 34, 35]. In order to better identify subpopulations of FEP patients who may benefit from targeted therapeutic measures, the aim of this study was to investigate prospectively the dynamics between insight and adherence throughout a 3-year specialized early psychosis program. Indeed it is now recognized that the level of insight is correlated with several decisive clinical outcomes for FEP patients, such as medication adherence [57] or global functioning [58].

The high rates of medication adherence reported in our study provide additional support to the benefit of specialized early intervention programs, when considering this is a critical component of the management of FEP. Indeed, such programs usually involve both an intensive treatment characterized by a collaborative and recovery-centered approach and make the patient an active partner in his therapeutic plan.

Medication adherence over the course of follow-up improved whether or not patients displayed insight at baseline. This result is of particular interest since poor adherence has been identified as one of the predominant unmet needs in the treatment of schizophrenia [59]. A recent systematic review of studies conducted in patients with psychotic spectrum disorders pointed young age, poor illness insight, cannabis use and positive symptomatology at baseline as risk factors for medication nonadherence, while antipsychotic medication adherence was associated with good illness insight and a positive attitude toward medication from both the patient and the family, and good illness insight were associated with satisfactory antipsychotic medication adherence [60]. A comprehensive review conducted in FEP patients found lack of insight among many other factors as a significant predictor of nonadherence, supporting the implementation of early intervention programs [17]. Finally, a recent study conducted in patients with schizophrenia explored the association between insight, therapeutic alliance, and perceived trauma related to psychiatric treatment [61, 62]. The results showed a positive correlation between medication adherence and insight or therapeutic alliance, while greater adherence was negatively correlated with perceived trauma. The authors hypothesized that interventions aimed at enhancing insight or reducing perceived trauma could promote better medication adherence. In this respect, the intensive and recovery centered treatment ideally provided by specialized early intervention programs facilitates the development of a therapeutic relationship [63].

Apart from strategies aimed at improving adherence to medication such as the use of long-acting injectable (LAI) antipsychotics [64, 65], motivational interviews, web-based psychoeducation, or SMS medication reminders [66], targeting insight seems to be useful to enhance adherence through a synergic process, which in turn improves outcome [67], particularly in terms of functional recovery. Furthermore, our finding that gain in adherence appears to occur earlier than improvement in insight throughout the program is interesting. This suggests that when the adjustment level at the end of follow-up reaches that of the premorbid period, it seems to depend rather on adherence and not on insight. It already has been shown that a strong therapeutic alliance has a subsequent positive effect on medication adherence and outcome [63]. However, this does not call into question the likely role of insight in enhancing medication adherence. Indeed, the development of a relationship of trust and respect between the patient and the clinician, which ultimately leads to a strong therapeutic alliance, takes place over several months or years for a certain category of patients. Insight could thus take longer to emerge but could have a greater reinforcing effect on medication adherence in the longer term. This hypothesis is in line with previous studies that showed that patients with high medication adherence see their level of insight increase over the course of follow-up [25].

Despite the methodological strength of our 3-year prospective study design, it faces a number of limitations. First, medication adherence was evaluated using a subjective assessment by the case manager, which relies on information from patients or relatives. Nevertheless, this remains the most widely used method in the literature [30, 68, 69]. Also, the rates of patients on LAI versus oral antipsychotics respectively were not included in the statistical models. Other factors that may have an impact on adherence were not considered in our analysis, such as cognitive and metabolic parameters, or unhealthy lifestyle, which are known to be affected in psychosis. Insight was assessed using a simple three-point Likert scale, possibly lacking precision. Nonetheless, other Likert-scale type instruments have shown good convergent and discriminant validity and reliability [70]. Inter-rater reliability was not determined, in relation to possible change of insight over time. Finally, patients’ attitudes toward medication were not considered. In this respect, one cross-sectional study looked at the impact of insight into illness and the global knowledge of patients with schizophrenia about their ongoing medication on their attitudes toward drug treatment [67]. The results concluded that good insight is associated with more favorable attitudes toward antipsychotic medication. In this regard, we assumed that attitudes toward medication and medication adherence were positively correlated.

In conclusion, this study adds support to the hypothesis that it is possible to identify latent subpopulations within a population of FEP patients based on the two variables of insight and medication adherence, which evolve dynamically during follow-up. We also have shown that adherence is the first variable to improve but that a gain in insight in the longer term possibly plays a role in the reinforcement of adherence. Finally, patients with a high level of adherence and to a lesser extent insight are those with the greatest chance of functional recovery. By showing potential benefits of individual treatment through specialized early intervention programs, these results reinforce the need for their development and implementation in patients with psychosis, with a specific attention to therapeutic strategies targeting medication adherence.

## Data Availability

The data that support the findings of this study are available on request from the corresponding author. The data are not publicly available due to privacy or ethical restrictions.
